# Inhibition of Aortic Intimal Hyperplasia and Vascular Smooth Muscle Proliferation and Extracellular Matrix Protein Expressions by Astragalus–Angelica Combination

**DOI:** 10.1155/2018/1508637

**Published:** 2018-08-13

**Authors:** Huifang Yan, Xiwei Peng, Hao Xu, Jiahuan Zhu, Changqing Deng

**Affiliations:** ^1^Molecular Pathology Laboratory, Hunan Provincial Key Laboratory for Prevention and Treatment of Integrated Traditional Chinese and Western Medicine on Cardio-Cerebral Diseases, Hunan University of Chinese Medicine, Changsha, Hunan 410208, China; ^2^Molecular Pathology Laboratory, Hunan University of Chinese Medicine, Changsha, Hunan 410208, China

## Abstract

VSMC proliferation and ECM deposition always resulted in intimal hyperplasia. Astragalus–Angelica combination has a protective effect on the cardiovascular system. The inhibition effect of different Astragalus–Angelica combination on the hyperplastic intima after vascular balloon injury in rats was investigated in this study. Astragalus–Angelica combination can inhibit the intima hyperplasia after balloon injury, in which a 1:1 ratio shows excellent results. Astragalus–Angelica combination can enhance the expression of smooth muscle α-actin (SMа-actin) and inhibit the expression of proliferating cell nuclear antigen (PCNA), cyclin D1, cyclin E, collagen I (Col-I), fibronectin (FN), and matrix metallopeptidase-9 (MMP-9) in hyperplastic intima, suggesting that Astragalus–Angelica combination can inhibit the intimal hyperplasia of blood vessels in rats. The mechanism is related to the inhibition of PI3K/Akt signaling pathway activation and thereby inhibits the phenotypic transformation and cell proliferation of VSMCs and thus inhibits the extracellular matrix (ECM) deposition of vascular wall during intimal hyperplasia.

## 1. Introduction

Astragalus and Angelica are important herbs of traditional Chinese medicine that are often used in combination for the treatment of many types of cardiovascular diseases. The most popular combination of Astragalus and Angelica is Dangguibuxuetang (Danggui Buxue Tang, DBT), which was created in 1247 by Li Dongyuan. DBT consists of five parts of Astragalus and one part of Angelica. In recent years, studies showed that the Astragalus–Angelica combination not only has the effect of promoting hematopoiesis [1], but also has a cardiovascular protective effect [2]. The serum containing Astragalus–Angelica combination decoction can protect the vascular endothelium by promoting the function of endothelial progenitor cells, and the effect is related to the regulation of the PI3K/Akt signaling pathway [3].

The basic pathological process of cardiovascular diseases such as atherosclerosis, vascular remodeling of hypertension, and vascular restenosis after intervention involves the proliferation and migration of VSMCs from the media to the intima, and synthesis of ECM, forming new intima and causing vascular stenosis [4]. Therefore inhibiting the proliferation of VSMCs after vascular endothelial injury and inhibiting the synthesis of ECM became important measures to attenuate the intimal hyperplasia [5]. Our study takes antiproliferation of VSMCs and antisynthesis of ECM as the breakthrough point and uses rat vascular intimal hyperplasia as a model to study on the inhibitory effect of the Astragalus–Angelica combination on vascular intimal hyperplasia.

## 2. Materials and Methods

### 2.1. Animal Model

Healthy male Sprague–Dawley (SD) rats, weighing 220-250 g, SPF, and 8 weeks old, were provided by the Experimental Animal Center at the Hunan University of Chinese Medicine. Animals were allowed to drink and eat freely and caged in an environment of 25°C and 45%-65% relative humidity. The animal protocols were approved by the Animal Ethics Committee of Hunan University of Chinese Medicine, certificate number: 43004700027218, experimental animal license number: SCXK (Hunan) 2013-0004. The disposal of animals during the experiment was consistent with “Guidance Suggestions for the Care and Use of Laboratory Animals” from the Ministry of Science and Technology of China.

A model of intimal hyperplasia of thoracoabdominal aorta was established by balloon catheter injury according to our previously modified method [6]. The rats were anesthetized and an incision was made on the left midline of the neck to isolate the left common carotid artery. The balloon catheter was inserted into the left common carotid artery through the aortic arch down to abdominal aorta with a depth of about 6–7cm. After pressurized with a balloon pump the balloon catheter was pulled back and forth from the abdominal aorta to the aortic arch for four times. After the balloon was pumped back to the negative pressure and the catheter was withdrawn from the artery, the common carotid artery was ligated at the proximal end to stop bleeding. The muscle, subcutaneous tissue, and skin were sutured separately after an appropriate amount of gentamicin is applied to the wound followed by suture. Penicillin (200,000 units) was injected intraperitoneally at the end of the operation and in the following 2 days. The animals with a failed operation were excluded and 8–10 rats in each group were used for next experimental procedures.

### 2.2. Chinese Herbal Decoction Preparation and Positive Control Drug

Astragalus is the dry root of* Astragalus membranaceus* (Fisch.) Bge. var. mongholicus (Bge.) Hsiao, which originated from Inner Mongolia. Angelica is the dry root of* Angelica sinensis* (Oliv.) Diels, which originated from Gansu. All the herbs were provided and identified by the Pharmacy Department of Hunan University of Chinese Medicine (Herbs Appraiser: Professor Ya-jie Zuo), and the voucher specimens were stored at the Affiliated Hospital Herbarium, Hunan University of Chinese Medicine.

Astragalus, Angelica, and combinations of Astragalus–Angelica in different ratios (5:1, 2:1, 1:1, 1:2, 1:5) were prepared. The crude herbs of Astragalus and Angelica were weighed according to the ratios, chopped, and placed into the leacher for extraction three times using the water reflux method and the final concentrations of Astragalus, Angelica, and different combinations were 0.22 g/mL, 0.11 g/mL, and 0.39 g/mL, respectively. Following 0.1% of sodium benzoate addition, the concentrate was subpackaged and stored at −4°C.

Calycosin glycoside, calycosin, formononetin, and astragaloside IV are the characteristic components of Astragalus, and ferulic acid is the characteristic component of Angelica [7]. Therefore, the components were measured to identify Astragalus and Angelica by ultra performance liquid chromatography tandem mass spectrometry (UPLC-MS/MS) by our research team previously on a Waters-Xevo-G2-S QTof UPLC-MS/MS instrument (Acquity system, V4.1 Masslynx chromatography workstation, Waters Co., USA) [8]. The contents of effective components in Astragalus extract were as follows: Astragalus calycosin glycoside 462.96 *μ*g/g crude herb, calycosin 351.62 *μ*g/g crude herb, formononetin 52 *μ*g/g crude herb, and astragaloside 33.38 *μ*g/g crude herb. The contents of the active constituents in the extract of Angelica were as follows: ferulic acid 328.74 *μ*g/g crude herb. The contents of effective components in the extract of Astragalus–Angelica 1:1 compatibility are ferulic acid 438.86 *μ*g/g crude herb, Astragalus calycosin glycoside 438.86 *μ*g/g crude herb, calycosin 267.82 *μ*g/g crude herb, formononetin 44.34 *μ*g/g crude herb, and astragaloside 12.46 *μ*g/g crude herb [8]. Studies have shown that the five chemical components can be absorbed into the plasma [9] through small intestine [10] and are related to vascular remodeling factors. Both ferulic acid and calycosin-7-glucoside could improve endothelium dysfunction in dose dependence [11]. Formononetin has a suppressive effect on PDGF-BB-stimulated VSMCs proliferation and migration, which occur partly via the inhibition of Akt signaling pathway [12]. Astragaloside IV inhibits PDGF-BB-stimulated VSMC proliferation and migration by inhibiting the activation of the p38 MAPK signaling pathway [13]. Ferulate can inhibit the proliferation of SMC and the inhibitory effects are related to the inhibition of lipid peroxidation [14]. Calycosin protected HUVEC from LPS-induced endothelial injury through suppression of Rho/ROCK pathway and regulation of Akt pathway [15]. However, the effect of such combination of pure compounds for the effect of VSMC and ECM has not been reported.

Atorvastatin calcium can not only reduce blood lipids but also play a cardiovascular protective role through antioxidant, anti-inflammatory, and vascular endothelium protection; it inhibits the intimal hyperplasia after vascular endothelial injury [16]. Therefore, atorvastatin calcium produced by Neo-Dankong Pharmaceutical Co., Ltd. (Batch number: 20160803, specification: 10 mg/ tablet) was used as the positive control drug and was suspended in distilled water (concentration: 1mg/mL) before use.

### 2.3. Balloon Catheter

A 2.0 × 15 mm Runjin medical balloon catheter, a Runthrough guide-wire, and a medical balloon pressure pump are all manufactured by Japanese Terumo Corporation, and the three parts were assembled before the experiment.

### 2.4. Reagents

Primary antibody was rabbit anti-rat polyclonal antibodies (beta-actin, SMа-actin, PCNA, cyclin E, Col-I, FN, MMP-9, tissue inhibitor of metalloproteinases-1 (TIMP-1), pan-Akt (phospho T308), and rabbit anti-rat cyclin D1 monoclonal antibody [EPR2241]) which were purchased from Abcam and rabbit anti-rat phospho PI 3 kinase p85 (phospho Tyr467)/p55 (phospho Tyr199) polyclonal antibody which was purchased from GeneTex. Secondary antibody was Horseradish Peroxidase-conjugated AffiniPure Goat Anti-Rabbit IgG (H+L) (Jackson, 111-035-003). PV-9000 Polymer Detection System For Immuno-Histological Staining (ZSGB-BIO, Beijing, China, PV-9000), bicinchoninic acid protein assay kit (Beyotime Biotechnology, Shanghai, China, P0010), and sodium dodecyl sulphate-polyacrylamide gel electrophoresis (SDS-PAGE) Gel Kit (Beyotime Biotechnology, Shanghai, China) were also used.

### 2.5. Grouping and Administration Methods

The baseline geometric design method [17] was used and the baseline combination of Astragalus–Angelica was set at 5:1, and the Astragalus and Angelica dosage was increased or decreased by 16.6% on both sides in order to design different combinations of Astragalus–Angelica, in addition with the positive drug atorvastatin group (10 mg/kg). Sprague–Dawley rats were randomly divided into normal group (the left common carotid artery was exposed and ligated, but the balloon was not used), model group (the model was given normal saline with the same volume of the drug group by intragastric administration), Astragalus and Angelica with single use or different ratios groups, and atorvastatin group. Drugs or water (in normal group and model group) was administrated by gavage from the first day after operation and one time a day for 14 days.

### 2.6. Morphology Analysis of Intimal Hyperplasia

We used our previously established vascular morphometry testing method [6]. After chloral hydrate anesthesia at the fifteenth day after operation, the rats were bled to death and the abdominal aortas were collected. The vessels used for Western blot detection are placed directly into the cryopreservation tube and stored at -80°C. The vessels used for Masson staining and immunohistochemistry were fixed with 4% paraformaldehyde at 4°C for 24h, dehydrated with ethanol gradient, vertically embedded with paraffin, and stored at 4°C.

Each segment of the vessel was cut intermittently for eight slices and stained with Masson stain. The results were observed under light microscopy with magnification of 100-400 times. MIAS medical image analysis system was used to take photos, and the inner area of the outer elastic membrane, inner elastic membrane, and lumen was measured by Image-Pro Plus 6.0 image analysis software. Based on the aforementioned results, the formula for calculation is as follows:

(1) Media  AreaMA=Inner  area  of  the  outer  elastic  membrane−Inner  area  of  the  inner  elastic  membraneIntimal  AreaIA=Inner  area  of  the  inner  elastic  membrane−Lumen  areaMedia  ThicknessMT=Inner  area  of  the  outer  elastic  membraneπ−Inner  area  of  the  inner  elastic  membraneπIntimal  ThicknessIT=Inner  area  of  the  outer  elastic  membraneπ−Lumen  areaπHyperplasia  Ratio  of  Intimal  AreaHRIA=IAIA+MA×100%Hyperplasia  Ratio  of  Intimal  AreaHRIA=ITIT+MT×100%

### 2.7. Immunohistochemical Staining Analysis

Immunohistochemical staining was performed according to the kit instructions: After 3% hydrogen peroxide treatment and microwave antigen repairmen, add 1:100 diluted rabbit anti-rat polyclonal antibodies (SMа-actin, PCNA, Col-I, FN) 50 *μ*L per section and incubate overnight at 4°C, then do washing three times with phosphate buffered saline (PBS) and incubation with Polymer Helper for 30 min at 37°C, then washing three times with PBS again and incubation with polyperoxidase-anti-mouse/rabbit immunoglobulin IgG for 30 min at 37°C. Subsequently, the sera were washed three times with PBS and subjected to coloration with DAB, wash, counterstaining with hematoxylin, followed by dehydration, clearing, and mounting. Under light microscopy, the positive expression was brownish yellow punctate or fibrous staining, concentrated in the cell membrane, cytoplasm, or intercellular space. No brownish yellow staining was found in the negative controls. MIAS medical image analysis system was used to take photos, and three different visual fields were selected from each slice; subsequently, integrated optical density (IOD) was measured by Image-Pro Plus 6.0 in order to reflect the expression intensity of the target protein.

### 2.8. Western Blot Analysis

The vascular tissue was cut as much as possible, and placed in a glass homogenizer on ice and then homogenized with 1 mL of cell lysate and 2 *μ*L of PMSF. After 0.5 h resting on ice and 10 min centrifuging at 4°C 10,000 r/min, the supernatant was collected and the total protein concentration was determined by a BCA kit. The total proteins were separated by 12% SDS-PAGE and transferred to a PVDF membrane.

The membrane was blocked in 5% PBS-skim milk for 1.5 h at room temperature and incubated for overnight at 4°C with 1:1000 dilution of rabbit anti-rat polyclonal antibodies (beta-actin, cyclin E, MMP-9, TIMP-1, phospho PI 3 kinase p85 (phospho Tyr467) /p55(phospho Tyr199), pan-Akt (phospho T308)) or 1:1000 dilution of rabbit anti-rat cyclin D1 monoclonal antibody. The antibody treated membrane was washed for 3×10 min with PBS and then incubated with a 1: 1000 dilution of peroxidase-conjugated secondary antibody for 1.5 h at room temperature. The membrane was washed again for 3×10 min with PBS and added with Western Bright ECL-HRP Substrate and was photographed in the gel imaging system. The IOD of the target band was determined by Image-Pro Plus 6.0, and the relative expression of the target protein was determined by the IOD ratio of the target band to the beta-actin band.

### 2.9. Statistical Analysis

SPSS 17.0 statistical software was used for analysis, and the experimental data were expressed by mean ± standard deviation ($\overset{-}{x}$±s), and one-way analysis of variance was used to compare the mean of each group.

Single-factor fuzzy comprehensive evaluation method was used to analyze the therapeutic effect of Astragalus and Angelica in different proportions [18]. By determining the evaluation factor set* X=*(x_1_, x_2_,…, x_m_) and the judgement set* Y=*(y_1_, y_2_,…, y_m_), if the closing grade C_j_(*Y*_j_,* X)* is the maximum degree of closeness between n fuzzy sets* Y*_1_,* Y*_2_,…,* Yn*, then* X,Y*_j_, and* X* are considered to be the closest to each other. TOPSIS method is commonly used to judge the evaluation factor set X as the principle of closeness degree.

## 3. Results

### 3.1. Effect of Different Combinations of Astragalus and Angelica on Intimal Hyperplasia

A vascular morphological study showed that the intima of the vascular endothelium in the normal group was intact, showing a single layer without hyperplasia. The intima of the model group showed homogeneous or heterogeneous thickening, and a large number of hyperplastic VSMCs existed, disorder arranged, and the lumen showed concentric or eccentric stenosis, with obvious intimal hyperplasia.

**Figure 1:**
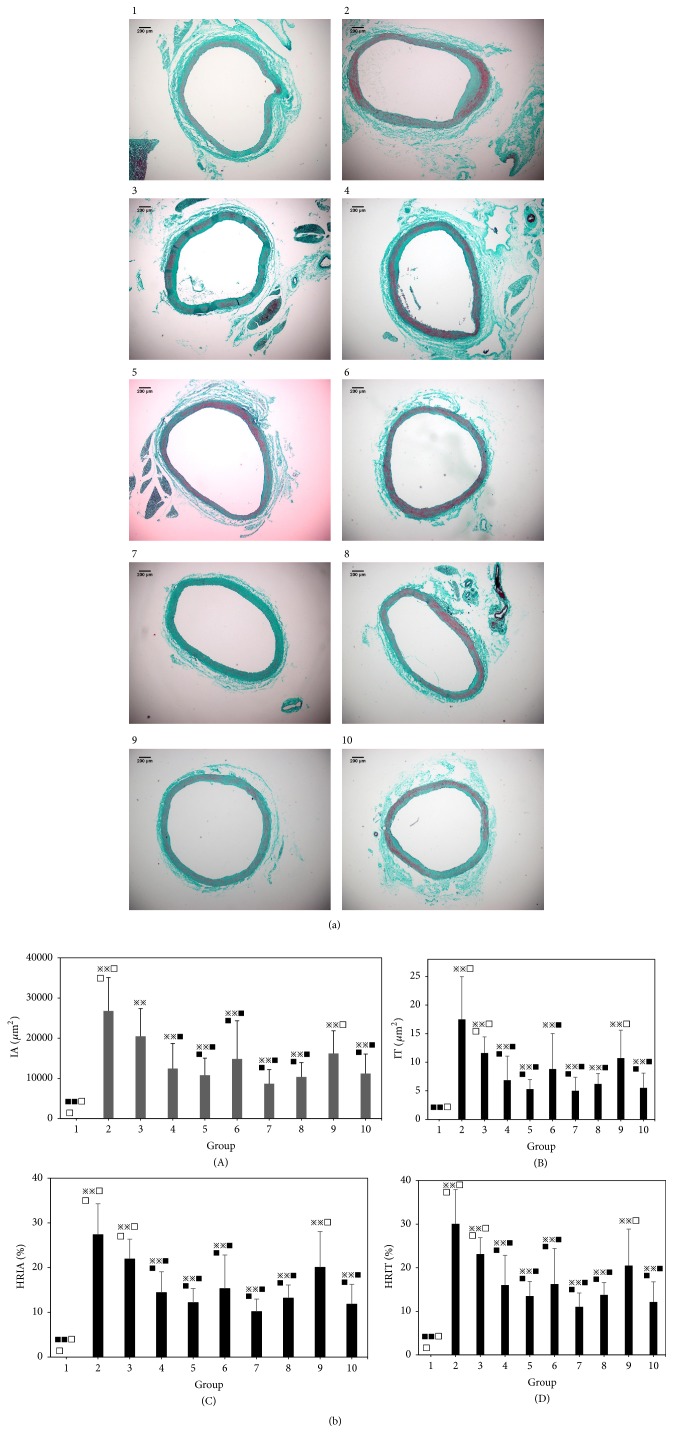
Comparison of intimal hyperplasia among the groups. (a) Pathological morphology of blood vessels (Masson staining, ×100, Bar=200*μ*m). (b) Comparison of morphometric indexes of vessels among the groups ($\overset{-}{x}$±s, n=8). 1. Normal. 2. Model. 3. Astragalus (2.17g/kg). 4. Angelica (1.08g/kg). 5. Astragalus–Angelica 1:2 (Astragalus 1.3g/kg + Angelica 2.6g/kg). 6. Astragalus–Angelica 1:5 (Astragalus 0.65g/kg + Angelica 3.25g/kg). 7. Astragalus–Angelica 1:1 (Astragalus 1.95g/kg + Angelica 1.95g/kg). 8. Astragalus–Angelica 5:1 (Astragalus 3.25g/kg + Angelica 0.65g/kg). 9. Astragalus–Angelica 2:1 (Astragalus 2.6g/kg + Angelica 1.3/kg). 10. Atorvastatin (10mg/kg). (A) Intimal area (IA). (B) Intimal thickness (IT). (C) Hyperplasia ratio of intimal area (HRIA). (D) Hyperplasia ratio of intimal thickness (HRIT). ^*※*^*P*<0.05, ^*※※*^*P*<0.01 versus normal group; ^■^*P*<0.05, ^■■^P< 0.01 versus model group; ^□^*P*<0.05, ^□□^*P*<0.01 versus Atorvastatin group.

The change of intimal hyperplasia in single Astragalus group and Astragalus–Angelica 2:1 group was the same as that in the model group. The vascular intima of the single Angelica group, Astragalus–Angelica 1:5, Astragalus–Angelica 1:2, Astragalus–Angelica 1:1, Astragalus–Angelica 5:1, and atorvastatin groups also showed proliferative changes, but the severity of hyperplasia was alleviated compared with the model group. See Figure 1(a).

Morphometric analysis of the vessels showed that compared with the normal group, the IA, IT, HRIA, and HRIT of the model group were significantly increased (P<0.01), but the MA and MT showed no significant change (P > 0.05). Compared with the model group, the vascular tissue IA, IT, HRIA, and HRIT of single Angelica group, Astragalus–Angelica 1:1, Astragalus–Angelica 1:2, Astragalus–Angelica 1:5, Astragalus–Angelica 5:1, and atorvastatin groups were significantly decreased (P < 0.01 or P < 0.05), but Astragalus–Angelica 2:1 and single Astragalus group showed no significant change (P > 0.05). However, there was no significant change in MA and MT in each group (P > 0.05). Compared with the atorvastatin group, there was no significant change in IA, IT, HRIA, and HRIT of single Angelica group, Astragalus–Angelica 1:1, Astragalus–Angelica 1:2, Astragalus–Angelica 1:5, and Astragalus–Angelica 5:1 groups (P > 0.05), and the IA, IT, HRIA, HRIT of single Astragalus group and Astragalus–Angelica 2:1 were significantly higher than those in the atorvastatin group (P < 0.01). In different combinations of Astragalus and Angelica, in the Astragalus–Angelica 1:1 group, the decrease in IA, IT, HRIA, and HRIT was the most significant. See Figure 1(b).

Single-factor fuzzy comprehensive evaluation shows that the closing grade C value of the Astragalus–Angelica 1:1 group and atorvastatin group was the highest, and the curative effect was the best. The closing grade C value of single Astragalus group and that of the Astragalus–Angelica 1:1 group were the same, and the lowest in each group, and the closing grade C values of single Angelica group and Astragalus–Angelica 1:2, Astragalus–Angelica 1:5, and Astragalus–Angelica 5:1 group were in the middle. The results show that there were no obvious effects on vascular intimal hyperplasia of single Astragalus group and Astragalus–Angelica 2:1 group, and there were certain antivascular intimal hyperplasia effect of single Angelica group, Astragalus–Angelica 1:2, Astragalus–Angelica 1:5, and Astragalus–Angelica 5:1, and the strongest antivascular intimal hyperplasia effect was with Astragalus–Angelica 1:1. See Table 1.

### 3.2. Effect of Different Combinations of Astragalus and Angelica on Smooth Muscle *α*-Actin Expression in the Intima of Proliferative Vessels

**Figure 2:**
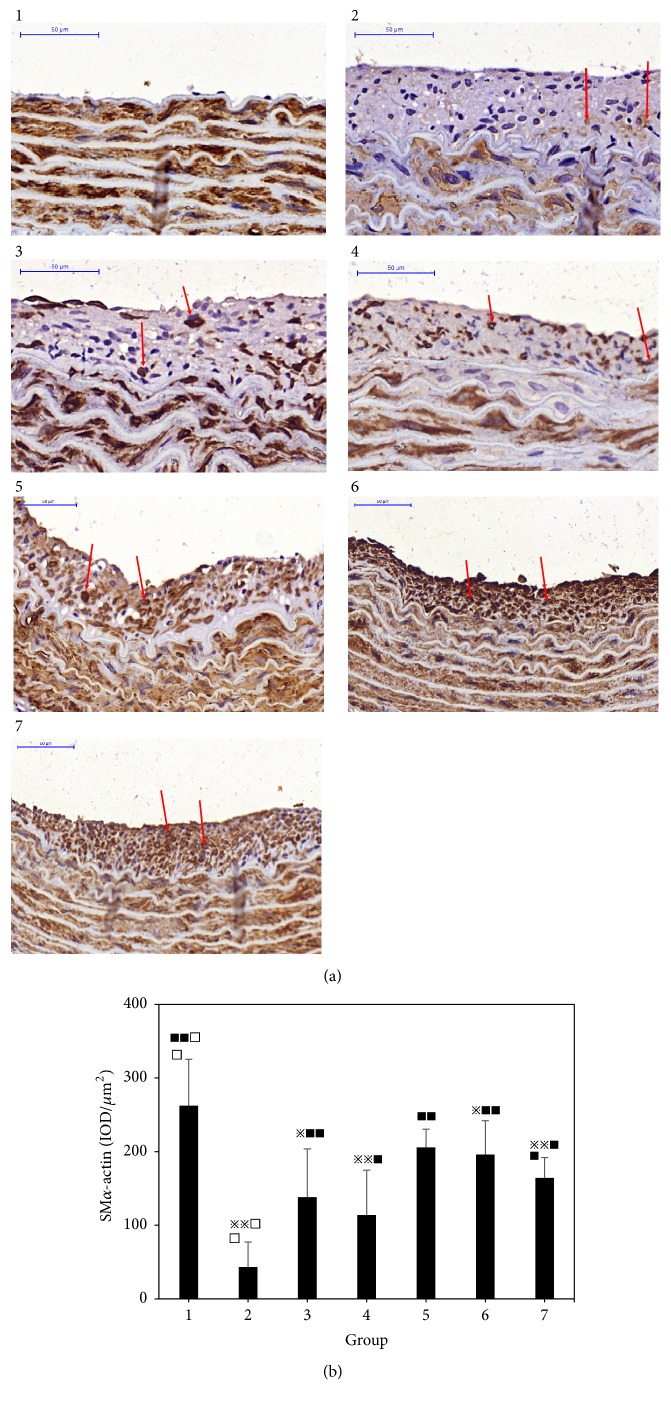
Expression of smooth muscle *α*-actin in the intima of proliferative vessels. (a) Immunohistochemical results of smooth muscle *α*-actin (×400, Bar = 50*μ*m). (b) Comparison of smooth muscle *α*-actin expression among the groups ($\overset{-}{x}$±*s*,* n*=5). 1. Normal. 2. Modelgroup. 3. Astragalus. 4. Angelica. 5. Astragalus–Angelica1:1. 6. Astragalus–Angelica5:1. 7. Atorvastatin. ^*※*^*P*<0.05, ^*※※*^*P*<0.01 versus normal group; ^■^*P*<0.05, ^■■^P< 0.01 versus model group; ^□^*P*<0.05, ^□□^*P*<0.01 versus Atorvastatin group.

Compared with the normal group, the expression of VSMC contraction phenotype marker smooth muscle *α*-actin in the intima of the vascular hyperplasia in the model group was significantly decreased (P <0.01). Compared with the model group, the expression of smooth muscle *α*-actin in the intimal hyperplasia of the vascular hyperplasia in single Angelica or Astragalus group, Astragalus–Angelica 1:1, Astragalus–Angelica 5:1, and atorvastatin groups was significantly increased (P <0.01 or P < 0.05). Compared with the atorvastatin group, there were no significant differences in the expression of smooth muscle *α*-actin in the intima of vascular hyperplasia in single Angelica or Astragalus group, Astragalus–Angelica 1:1, and Astragalus–Angelica 5:1 groups (P >0.05). See Figure 2.

### 3.3. Effect of Different Combinations of Astragalus and Angelica on PCNA Expression in the Intima of Proliferative Vessels

**Figure 3:**
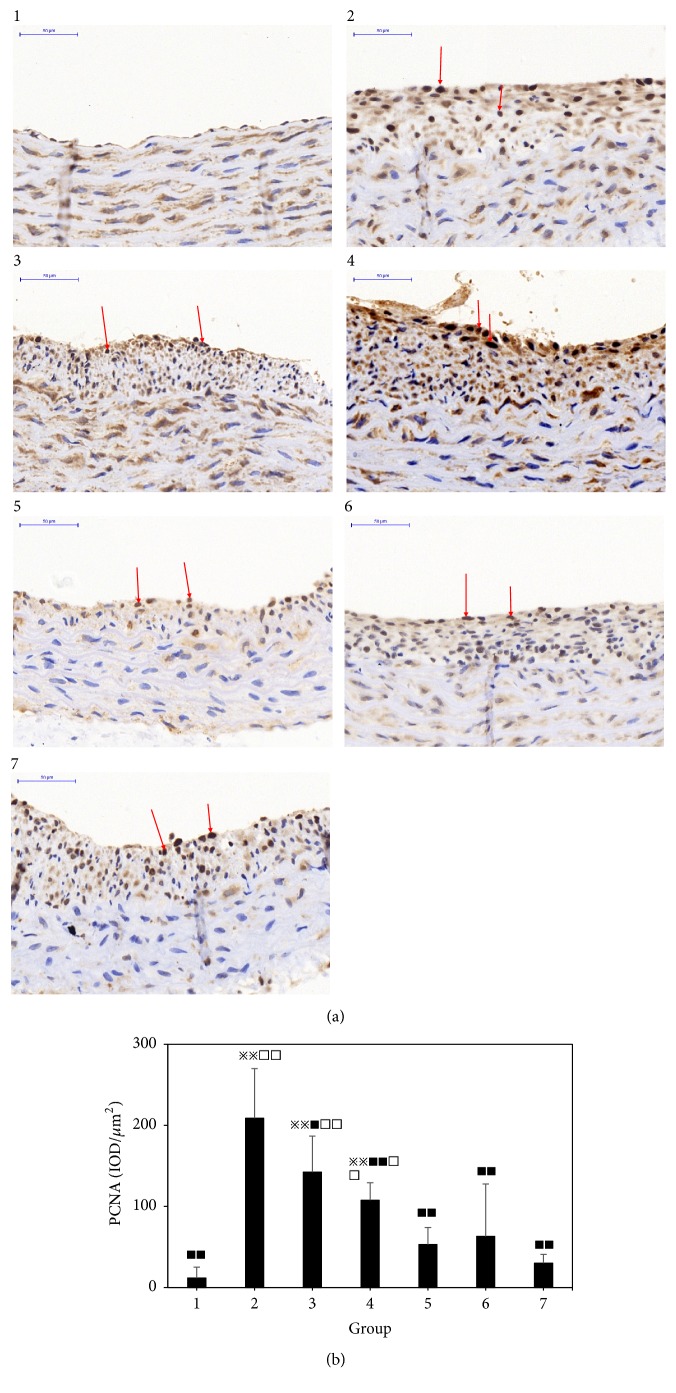
Expression of PCNA in the intima of proliferative vessels. (a) Immunohistochemical results of PCNA (×400, Bar = 50*μ*m). (b) Comparison of PCNA expression among the groups ($\overset{-}{x}$±*s*,* n*=6) 1. Normal. 2. Modelgroup. 3. Astragalus. 4. Angelica. 5. Astragalus–Angelica1:1. 6. Astragalus–Angelica5:1. 7. Atorvastatin. ^*※*^*P*<0.05, ^*※※*^*P*<0.01 versus normal group; ^■^*P*<0.05, ^■■^P< 0.01 versus model group; ^□^*P*<0.05, ^□□^*P*<0.01 versus Atorvastatin group.

Compared with the normal group, the expression of PCNA in the intima of the vascular hyperplasia in the model group was significantly increased (P <0.01). Compared with the model group, the expression of PCNA in the intimal hyperplasia of the vascular hyperplasia in single Angelica or Astragalus group, Astragalus–Angelica 1:1, Astragalus–Angelica 5:1 and atorvastatin groups was significantly decreased (P <0.01 or P < 0.05). Compared with the atorvastatin group, there were no significant differences in the expression of PCNA in the intima of vascular hyperplasia in Astragalus–Angelica 1:1 and Astragalus–Angelica 5:1 groups (P >0.05). See Figure 3.

### 3.4. Effect of Different Combinations of Astragalus and Angelica on Vascular Cell Cycle-Related Proteins Cyclin D1 and Cyclin E Expression in the Intima of Proliferative Vessels

**Figure 4:**
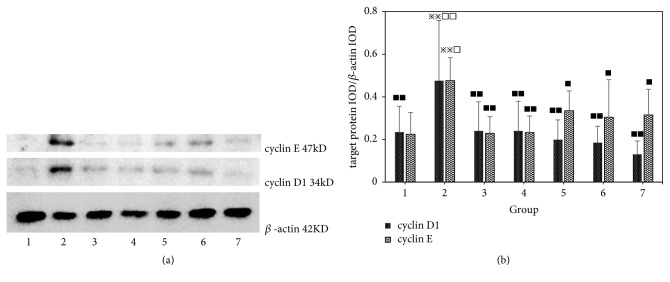
Comparison of cell cycle-related proteins cyclin D1 and cyclin E expressions in vascular tissues among the groups ($\overset{-}{x}$±*s*,* n*=6). (a) Western-blotting patterns of cyclin D1 and cyclin E protein. (b) Comparison of cyclin D1 and cyclin E protein expressions: 1. Normal. 2. Model. 3. Astragalus. 4. Angelica. 5. Astragalus–Angelica1:1. 6. Astragalus–Angelica5:1. 7. Atorvastatin. ^*※*^*P*<0.05, ^*※※*^*P*<0.01 versus normal group; ^■^P<0.05, ^■■^P< 0.01 versus model group; ^□^*P*<0.05, ^□□^*P*<0.01 versus Atorvastatin group.

Compared with the normal group, the expression of cyclin D1 and cyclin E in the intima of the vascular hyperplasia in the model group was significantly increased (P <0.01). Compared with the model group, the expression of cyclin D1 and cyclin E in the intimal hyperplasia of the vascular hyperplasia in single Angelica or Astragalus group, Astragalus–Angelica 1:1, Astragalus–Angelica 5:1, and atorvastatin groups was significantly decreased (P <0.01 or P < 0.05). Compared with the atorvastatin group, there were no significant differences in the expression of cyclin D1 and cyclin E in the intima of vascular hyperplasia in single Angelica or Astragalus group, Astragalus–Angelica 1:1, and Astragalus–Angelica 5:1 groups (P >0.05). See Figure 4.

### 3.5. Effect of Different Combinations of Astragalus and Angelica on Extra Cellular Matrix (ECM) Proteins Col-I and FN Expression in the Intima of Proliferative Vessels

**Figure 5:**
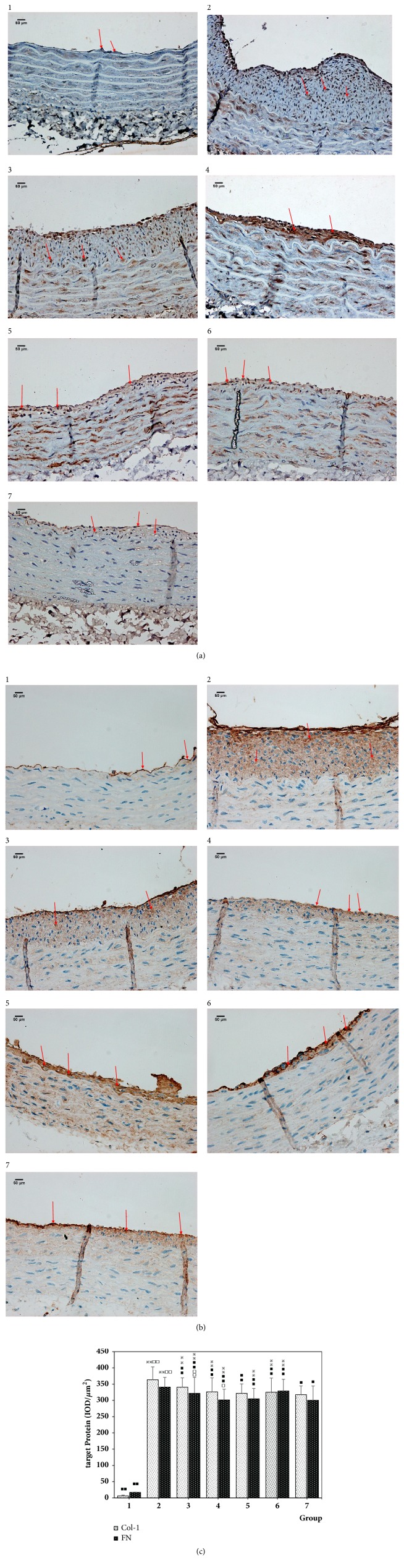
Expression of Col-I and FN in the intima of proliferative vessels. (a) Immunohistochemical results of Col-I (× 400, Bar = 50*μ*m). (b) Immunohistochemical results of FN (× 400, Bar = 50*μ*m). (c) Comparison of Col-I and FN expression among the groups ( ± s, n = 8) 1. Normal. 2. Model group. 3. Astragalus. 4. Angelica. 5. Astragalus–Angelica 1:1. 6. Astragalus–Angelica 5:1. 7. Atorvastatin. ^*※*^P < 0.05, ^*※※*^P < 0.01 versus normal group; ^■^P < 0.05, ^■■^P < 0.01 versus model group; ^□^P < 0.05, ^□□^P < 0.01 versus Atorvastatin group.

Compared with the normal group, the expression of Col-I and FN in the intima of the vascular hyperplasia in the model group was significantly increased (P <0.01). Compared with the model group, the expression of Col-I and FN in the intimal hyperplasia of the vascular hyperplasia in single Angelica or Astragalus group, Astragalus–Angelica 1:1, Astragalus–Angelica 5:1, and atorvastatin groups was significantly decreased (P <0.01). Compared with the atorvastatin group, there were no significant differences in the expression of Col-I in the intima of vascular hyperplasia in single Angelica or Astragalus group, Astragalus–Angelica 1:1, and Astragalus–Angelica 5:1 groups (P >0.05), and there were no significant differences in the expression of FN in the intima of vascular hyperplasia in Astragalus–Angelica 1:1 group, and the expression of FN in the intimal hyperplasia of the vascular hyperplasia in single Angelica or Astragalus group and Astragalus–Angelica 5:1 groups was significantly increased (P <0.01 or P < 0.05). See Figure 5.

### 3.6. Effect of Different Combinations of ECM Regulatory Factors Expression in the Intima of Proliferative Vessels

**Figure 6:**
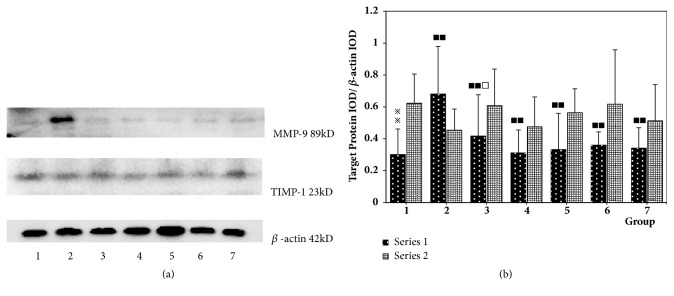
Comparison of MMP-9 and TIMP-1 protein expressions in vascular tissues among the groups ($\overset{-}{x}$±*s*,* n*=6). (a) Western-blotting patterns of MMP-9 and TIMP-1 protein. (b) Comparison of MMP-9 and TIMP-1 protein expressions: 1. Normal. 2. Model. 3. Astragalus. 4. Angelica. 5. Astragalus–Angelica1:1. 6. Astragalus–Angelica5:1. 7. Atorvastatin. ^*※*^*P*<0.05, ^*※※*^*P*<0.01 versus normal group; ^■^P<0.05, ^■■^P< 0.01 versus model group; ^□^*P*<0.05, ^□□^*P*<0.01 versus Atorvastatin group.

Compared with the normal group, the expression of MMP-9 in the intima of the vascular hyperplasia in the model group was significantly increased (P <0.01). Compared with the model group, the expression of MMP-9 in the intimal hyperplasia of the vascular hyperplasia in single Angelica or Astragalus group, Astragalus–Angelica 1:1, Astragalus–Angelica 5:1, and atorvastatin groups was significantly decreased (P <0.01 or P < 0.05). Compared with the atorvastatin group, there were no significant differences in the expression of MMP-9 in the intima of vascular hyperplasia in the single Angelica group, Astragalus–Angelica 1:1, and Astragalus–Angelica 5:1 groups (P >0.05), and the expression of MMP-9 in the intima of the vascular hyperplasia in the single Astragalus group was significantly increased (P <0.01). There were no significant differences in the expression of TIMP-1 in the intima of vascular hyperplasia among each group (P > 0.05). See Figure 6.

### 3.7. Effect of Different Combinations of Astragalus and Angelica on Akt, p-Akt, PI3K, and p-PI3K Expression in the Intima of Proliferative Vessels

Compared with the normal group, there were no significant differences in the expression of Akt and PI3K protein in the injured vessels of the model group (P >0.05). Compared with the model group, there were no significant differences in the expression of Akt and PI3K protein in the injured vessels of single Angelica or Astragalus groups, Astragalus–Angelica 1:1, Astragalus–Angelica 5:1, and atorvastatin groups (P >0.05). There were no significant differences in the expression of Akt and PI3K protein between the drug groups (P >0.05).

**Figure 7:**
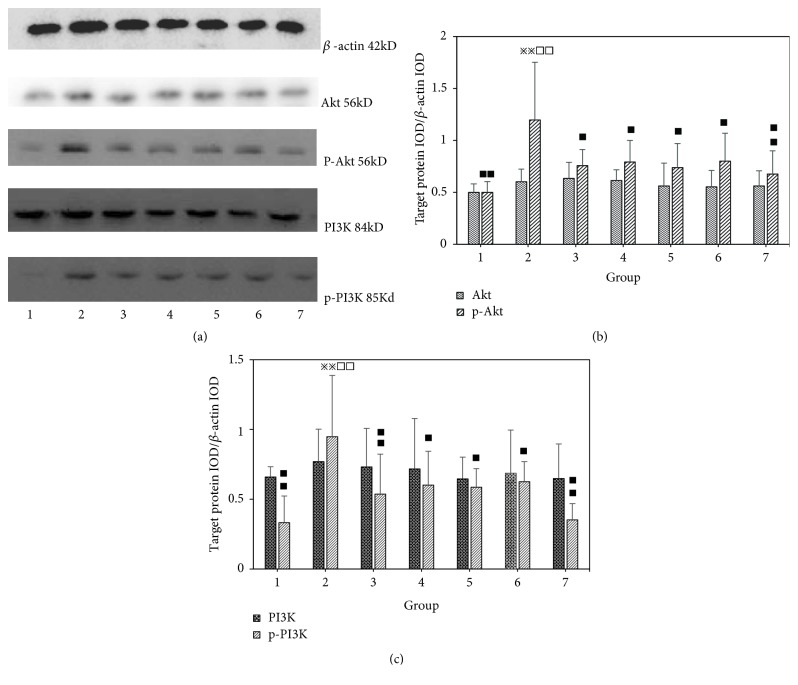
Comparison of Akt, p-Akt, PI3K, and p-PI3K protein expressions in vascular tissues among the groups ($\overset{-}{x}$±*s*,* n*=6). (a) Western-blotting patterns of Akt, p-Akt, PI3K, p-PI3K protein. (b) Comparison of Akt, p-Akt protein expressions. (c) Comparison of PI3K, p-PI3K protein expressions: 1. Normal. 2. Model. 3. Astragalus. 4. Angelica. 5. Astragalus–Angelica1:1. 6. Astragalus–Angelica5:1. 7. Atorvastatin. ^*※*^*P*<0.05, ^*※※*^*P*<0.01 versus normal group; ^■^P<0.05, ^■■^P< 0.01 versus model group; ^□^*P*<0.05, ^□□^*P*<0.01 versus Atorvastatin group.

Compared with the normal group, the expression of p-Akt and p-PI3K protein in the injured vessels of the model group was significantly increased (P <0.01). Compared with the model group, the expression of p-Akt and p-PI3K protein in the injured vessels in single Angelica or Astragalus group, Astragalus–Angelica 1:1, Astragalus–Angelica 5:1, and atorvastatin groups was significantly decreased (P <0.01 or P < 0.05). Compared with the atorvastatin group, there were no significant differences in the expression of p-Akt and p-PI3K protein in the injured vessels of single Angelica or Astragalus group, Astragalus–Angelica 1:1, and Astragalus–Angelica 5:1 groups (P >0.05). See Figure 7.

## 4. Discussion

Restenosis is a major and serious complication of PCI. Studies have shown that the proliferation of smooth muscle cells and the interaction of ECM and VSMCs are the main mechanism of restenosis after vascular intimal injury [19]. In the intimal hyperplasia disease, the proliferation and migration of VSMCs resulted in the accumulation of large amounts of ECM and deposition on the vascular wall, and the deposition of ECM promotes the proliferation and migration of VSMCs [20]. Thus, inhibition of the proliferation of VSMC and the deposition of ECM are important targets for preventing and treating of vascular proliferative lesions. Chinese medicine has many links regulating many targets; it may play an antiangiogenic effect by inhibiting the proliferation of VSMC and regulating the metabolism of ECM.

Vascular morphometric studies have shown that Astragalus–Angelica combination can decrease IA, IT, HRIA, and HRIT of the endothelial injury-induced vascular intimal hyperplasia in certain ratios of combinations, which suggested that the Astragalus–Angelica combination can inhibit intimal hyperplasia after vascular injury. Fuzzy comprehensive evaluation showed that Angelica played a dominant role in the antivascular intimal hyperplasia of Astragalus and Angelica combination, and Astragalus–Angelica 1:1 was the best combination. It is suggested that a proper proportion of Astragalus and Angelica should be adopted in the prevention and treatment of vascular intimal hyperplasia.

The phenotype of VSMCs is diverse and variable and Astragalus–Angelica combination could inhibit the phenotype transformation and proliferation of VSMCs [21]. When blood vessels are damaged or VSMCs are stimulated by growth factors in vitro, VSMCs were transformed from differentiated phenotype into dedifferentiated phenotype and acquired proliferative capacity. This process is called phenotypic modulation [22]. Vascular remodeling, which is caused by phenotypic modulation of VSMCs, is a key factor in the development of vascular restenosis and atherosclerosis [23], and smooth muscle *α*-actin is one of the most important markers of VSMC differentiated phenotype [24]. Our study showed that the expression of smooth muscle *α*-actin was decreased in the intimal hyperplasia of a vascular injury model, indicating VSMC modulation from differentiated to undifferentiated phenotype after vascular injury. Cell proliferation is activated by cell cycle, and the activation of cell cycle is the final common pathway of cell proliferation. PCNA is a nuclear protein involved in DNA synthesis related to cell proliferation and it is a nuclear polypeptide chain that can only be synthesized and expressed in proliferating cells [25]. Cyclin D1 and cyclin E play a key role in the regulation of cell cycle G0/G1 and Gl/S transformation, determining whether the cell is transformed from nonproliferative state to proliferative state [26].This study showed that the expression of PCNA, cyclin D1, and cyclin E increased in the intimal hyperplasia of the rat vascular injury model, indicating that the proliferation of the cells in the intima was enhanced after the vascular damage. Different Astragalus–Angelica combinations can increase the expression of smooth muscle *α*-actin and inhibit the expression of PCNA, cyclin D1, and cyclin E in the proliferative intima, suggesting that the inhibitory effect of Astragalus–Angelica combinations on intimal hyperplasia is mediated by the inhibition of VSMC phenotypic modulation and proliferation.

ECM plays an important role in maintaining the integrity of the vascular wall and the normal function of the vascular wall cells. It is generally believed that the enhanced expression of MMP-9 can promote the degradation of ECM, and the enhanced expression of TIMP can inhibit the degradation of ECM. This study shows that the balloon catheter can cause obvious intimal hyperplasia after injury of the vascular intima and the expression of Col-I and FN in proliferative intima increased, indicating that ECM synthesis increased in proliferative vascular intima, which further promoted VSMCs proliferation and intimal hyperplasia. The atorvastatin, single Astragalus, and Astragalus–Angelica 1:1 groups can inhibit the expression of Col-I and FN in hyperplastic endometrium, suggesting that the deposition of ECM in the hyperplasia endometrium can be inhibited, and thus the hyperplasia of endangium can be lightened. It is found that the single Angelica or Astragalus groups, Astragalus–Angelica 1:1, Astragalus–Angelica 5:1, and atorvastatin groups have a significant inhibitory effect on the expression of MMP-9, indicating that it can correct the imbalance of ECM clearance mechanism, inhibit the proliferation and migration of VSMCs, and stabilize plaque by inhibiting the expression of MMP-9. The increased expression of MMP-9 in the blood vessel did not lead to a significant change in the TIMP-1, which was associated with the disruption of the balance between MMP and TIMP by inducing the expression of MMP-9 gene and promoting the secretion of MMP-9 in inflammatory cells during vascular injury [27].

PI3K is involved in the biological effects of various growth factors. The PI3K/Akt pathway plays a vital role in cell growth and differentiation [28]. Activation of the PI3K/Akt signaling pathway is closely related to proliferation of vascular cell walls and inhibition of Akt activation in cells can inhibit the growth factor-induced growth of VSMCs [29]. Therefore, the activation of PI3K/Akt signaling pathway is related to VSMC phenotypic modulation and cell proliferation. This study showed that the expression of p-Akt and p-PI3K in the proliferative intima was significantly increased by balloon catheter injury to the intima and can be inhibited by single and combined use of Astragalus and Angelica, indicating that the activation of PI3K/Akt signaling pathway was associated with the proliferation of VSMC in the hyperplastic intima and Astragalus or Angelica and their combination can inhibit the proliferation of VSMC by inhibiting the activation of PI3K/Akt signaling pathway in the hyperplastic intima after vascular endothelial injury.

## 5. Conclusions

Balloon injury can induce VSMC phenotype transition and cell proliferation and increase ECM synthesis in intima, resulting in intimal hyperplasia. The Astragalus–Angelica combination plays an important role in inhibiting intimal hyperplasia by inhibiting the VSMC phenotypic modulation and proliferation as well as the synthesis of ECM in the intima, and the Astragalus–Angelica 1:1 combination is the best. The anti-intimal hyperplasia effect of Astragalus and Angelica combination is related to inhibition of the activation of PI3K/Akt signaling pathway, thereby inhibiting VSMC phenotype modulation and cell cycle transformation, and then plays the role of anti-VSMC proliferation and anti-ECM deposition. This reflects the regulation of multiple links and multiple targets in traditional Chinese medicine and provides an important basis for the use of Astragalus and Angelica combination in the prevention and treatment of intima proliferative diseases.

## Figures and Tables

**Table 1:** Analysis results of single factor fuzzy comprehensive evaluation.

Group	D^+^	D^−^	C	Ranking
Model group	0.502	0	0	8
Atorvastatin	0.045	0.465	0.912	2
Single Angelica group	0.106	0.396	0.789	5
Single Astragalus group	0.340	0.164	0.325	7
Astragalus–Angelica 1:2	0.085	0.417	0.831	3
Astragalus–Angelica 1:5	0.086	0.417	0.829	4
Astragalus–Angelica 1:1	0	0.502	1	1
Astragalus–Angelica 5:1	0.128	0.375	0.746	6
Astragalus–Angelica 2:1	0.340	0.164	0.325	7

Note: evaluation factor set *X *= {HRIA (%), HRIT (%)}, judgement set *Y *= {model group, Atorvastatin, single Angelica group, single Astragalus group, Astragalus–Angelica 1:2, Astragalus–Angelica 1:5, Astragalus–Angelica 1:1, Astragalus–Angelica 5:1, Astragalus–Angelica 2:1}. D value is vector distance.

## Data Availability

The data used to support the findings of this study are included within the article.
